# Genetic and Immunohistochemical Expression of Integrins ITGAV, ITGA6, and ITGA3 As Prognostic Factor for Colorectal Cancer: Models for Global and Disease-Free Survival

**DOI:** 10.1371/journal.pone.0144333

**Published:** 2015-12-16

**Authors:** Marcelo Moura Linhares, Renato José Affonso, Luciano de Souza Viana, Sandra Regina Morini Silva, Marcos Vinicius Araujo Denadai, Silvia Regina Caminada de Toledo, Delcio Matos

**Affiliations:** 1 Postgraduate Program in Interdisciplinary Surgery Science, Federal University of São Paulo UNIFESP-Escola Paulista de Medicina, São Paulo, Brazil; 2 Hospital de Cancer de Barretos-Fundação Pio XII, Barretos, Brazil; 3 Molecular Biology Laboratory, Federal University of São Paulo UNIFESP – EPM / GRAACC, São Paulo, Brazil; Thomas Jefferson University, UNITED STATES

## Abstract

**Objective:**

To evaluate the relationship between the expression profiles of 84 extracellular matrix (ECM) genes and the prognosis of patients with colorectal cancer (CRC).

**Methods:**

This retrospective study included 114 patients with stage I–IV CRC who underwent primary tumour resection. Quantitative real-time PCR and immunohistochemistry assays were conducted using primary tumour samples. Kaplan-Meier survival curves were also generated to identify differences in global survival (GS) and disease-free survival (DFS) for the hypo- or hyperexpression status of each marker. The log-rank test was used to verify whether the differences were significant. Stepwise Cox regression models were also used to identify the risk factors associated with GS and DFS in a multivariate mode, and then were used to score the risk of death associated with each marker, either independently or in association.

**Results:**

In the univariate analyses, significant differences in GS in relation to the expression profiles of ITGAV (p = 0.001), ITGA3 (p = 0.002), ITGA6 (p = 0.001), SPARC (p = 0.036), MMP9 (p = 0.034), and MMP16 (p = 0.038) were observed. For DFS, significant differences were observed in associated with ITGAV (p = 0.004) and ITGA3 (p = 0.001). However, only the ITGAV and ITGA6 gene markers for GS (hazard ratio (HR) = 3.209, 95% confidence interval (CI) = 1.412–7.293, p = 0.005 and HR = 3.105, 95% CI = 1.367–7.055, p = 0.007, respectively), and ITGA3 for DFS (HR = 3.806, 95% CI = 1.573–9.209, p = 0.003), remained in the final Cox regression models. A scoring system was developed to evaluate the risk of patient death based on the number of markers for the components of the final GS model. Scores of 0, 1, or 2 were associated with the following mean survival rates [CI]: 47.162 [44.613–49.711], 39.717 [35.471–43.964], 30.197 [24.030–36.327], respectively.

**Conclusions:**

Multivariate mathematical models demonstrated an association between hyperexpression of the ITGAV and ITGA6 integrins and GS, and also between the ITGA3 integrin and DFS, in patients with colorectal tumours. A risk scoring system based on detected hyperexpression of 0, 1, or 2 markers (e.g., ITGAV and/or ITGA6) was also found to accurately correlate with the GS curves generated for the present cohort.

## Introduction

Globally, colorectal carcinoma (CRC) is one of the most common malignant tumours diagnosed and is the third most common cause of death by neoplasm in the West. The prevalence of CRC is currently greater than 1,200,000 cases/year, and it is estimated that nearly 600,000 deaths are related to this disease. [1] Over the past twenty years a better understanding of the risk factors for developing this type of neoplasm has been gained, and this has been accompanied by improved preventative measures and the development of new drugs and surgical techniques. As a result, improved mortality rates related to CRC have been observed. [2,3]

CRC is treatable, and in most cases, is curable when it is detected in its early stages. [2] However, the average global five-year survival rate for all stages is 55% in developed countries, and 40% in developing countries. Furthermore, tumours diagnosed in the initial stages still pose a risk for systemic recurrence. [2,3]

To date, histopathological evaluation is the basis for a diagnosis, classification, and grading of CRC tumours. The grade of tumour penetration through the intestinal wall (the T stage), the presence and number of lymph nodes involved (the N stage), and the presence of distant metastasis (the M stage) are the most important prognostic factors, and these are also key factors in determining treatment strategy. Other factors that may contribute to a poor prognosis are the presence of undifferentiated tumours, mucinous subtype tumours, the presence of intestinal obstruction and/or perforation at the moment of diagnosis, and the presence of lymphovascular invasion. [4] Advances in our understanding of CRC biology, combined with a better understanding of the risk of recurrence and studies of patient survival curves, have improved the accuracy of patient classification. [4–7]

CRC is spread via direct invasion, generally through lymph canals or hematogenous routes. However, the intra- and intercellular signaling pathways that are responsible for the proliferation and survival of neoplastic cells are not fully characterized. [8–10] CRC appears to affect various pathways that regulate cell growth. Tumour growth and invasion also affect mechanisms of angiogenesis, epithelial growth factors, apoptosis, and remodeling of the extracellular matrix (ECM). [11]

Recent studies have shown that one of the mechanisms mediating tumour invasion and metastatic dissemination involves degradation of the ECM and the basal membrane, suggesting that interactions between the host and a tumour enhances the conditions for tumour dissemination. [12,13] The ECM consists of a complex network of macromolecules that are secreted by cells and they occupy the intercellular space, which includes the basal membrane, the blood matrix, and the conjunctive matrix. These macromolecules include different types of collagens, elastic system molecules, structural glycoproteins, glycosaminoglycans, and proteoglycans. [14] Thrombospondin, vascular cell adhesion molecule, 6A2 collagen, and metalloproteinases have been found to be directly involved in mediating cell proliferation, cell migration, and degradation of the ECM. [15–17] Overall, these macromolecules contribute to modulation of the ECM that occurs during both physiological and pathological processes.

Investigations of the cascade of events that are involved in the locoregional invasion and metastasis of CRC remain a great scientific challenge. The results of various studies have suggested that, in isolation, THBS1, VCAM-1, COL6A2, MMP1, and MMP16 genes and their respective molecules of expression—thrombospondin, vascular cell adhesion molecule, 6A2 collagen, and metalloproteinases—are involved in modulating the ECM during the process of CRC carcinogenesis. [11,15,18] The literature also contains a considerable amount of evidence with respect to genetic changes that are implicated in the rapid progression of CRC from its initial stages to its advanced stages. It is hypothesized that this process is initiated by anomalous signaling that activates genes to affect cancer cell dissemination and metastasis. [19,20]

The identification of molecules that undergo structural changes and their association with clinical and pathological stages could clarify the mechanisms involved in carcinogenesis. Furthermore, by recognizing the groups of genes involved, there is the potential for these genes to serve as markers of affected patients and prognosis. In this study, ECM genes in the tumour tissues of patients with colorectal adenocarcinoma were evaluated to identify potential correlations with TNM classification and survival data.

The objectives of this study are to identify a possible association between the expression of ECM genes in the tumour tissues of patients with CRC and the parameters of global survival (GS) and disease-free survival (DFS), as well as to build a predictive mathematical model of patient survival based on the hypo- or hyperexpression of these genes.

## Materials and Methods

### Patients

This study was reviewed and approved by the Research Ethics Committees of the Federal University of São Paulo (Protocol Number #2189/98) and Fundação Pio XII, Barretos (Protocol Number #178/2008), in accordance with Brazilian and international regulations for research with human subjects. All patients signed a written informed consent authorizing the use of biological material for the purpose of research.

Patients of both genders with synchronous colon and rectal cancer and that were older than 18 years of age were included in this study. Conversely, patients who received neoadjuvant treatment (e.g., chemotherapy or radiotherapy), patients without a primary CRC site, patients with a previous or current diagnosis of another primary malignancy in any location of the body other than non-melanoma skin cancer, patients with *in situ* carcinoma of the cervix, and patients with a known history of familial CRC were excluded. CRC patients had tumour samples collected, and the samples with the highest stages were selected for cryopreservation. Corresponding paraffin blocks were available for further histopathological analysis. Chromosomal (CIN) and microsatellite instability (MSI) status for these samples were not assessed.

### Gene expression analysis

Two pathologists from the Department of Pathological Anatomy independently reviewed the anatomopathological data from all of the selected cases. A total of 114 CRC cases underwent extraction of total RNA and cDNA synthesis. The reverse transcription reaction was performed using the SuperScript III First-Strand Synthesis SuperMix kit (Invitrogen, Carlsbad, CA, USA). Expression levels were then measured for each gene of interest and optimised for simultaneous use in the PCR Array platform using real-time PCR. A total of 84 ECM genes were detected using the Extracellular Matrix and Adhesion Molecules PCR Array (PAHS-013) (SABioscience, Qiagen, Valencia, CA, USA). Each plate analysed the expression of 84 genes related to the ECM, along with five endogenous controls (caretaker genes) including: one genomic DNA control, three controls for the reverse transcription reaction and three positive controls to test the efficiency of the PCR reaction.

Data analysis involved the ΔΔCt method using the following program: http://pcrdataanalysis.sabiosciences.com/pcr/arrayanalysis.php. The genes related to multifunctional ECM macromolecules, each with its own particularity, were analysed for hyperexpression and hypoexpression (e.g., fold change or odds ratio > 2), according to previously defined variables of interest. These genes were selected for further analysis of tissue expression.

### Construction of tissue microarray (TMA) blocks

Paraffin blocks were sectioned (4 μm thickness) and stained with hematoxylin-eosin. All sections were reviewed to confirm a diagnosis of CRC, and the histopathologic findings were reevaluated. A map was prepared using an Excel spreadsheet, and this contained the locations and identification of tissue samples that were used for the construction of each TMA block. The map also guided further readings of the IHC reactions. TMA blocks were prepared according to the manufacturer’s specifications (Beecher Instruments, Silver Spring, MD, USA) with the following steps: marking of the selected area in the respective paraffin block; creation of a hollow space in the recipient block; extraction of a cylindrical tissue from the donor block (measuring 1 mm in diameter and including the selected respective area of interest); transfer of the cylindrical tissue from the donor block to the hollow space created in the recipient block; insertion of the tissue in the block in fractions of millimeters. The resulting collection of tissue samples had a matrix arrangement. To assess the quality of each block for storage, the TMA blocks were each adhered onto a slide using an adhesive tape system (Instrumedics, Hackensack, N.J., USA). Samples were cut (4 um thickness), and a small roll was used to press the section on the tape. The tape with the attached histological section was then placed on a resin-coated slide (part of the adhesive system kit) and pressed with the same roll for better adherence. The slides were placed under UV light for 20 min and then were posteriorly exposed to a solvent solution (TPC) for another 20 minutes. After the slides were dried and the tapes were removed, the slides were embedded in paraffin and sent for storage under ideal cooling conditions.

### Immunohistochemical technique

Sections of TMA blocks were mounted onto glass slides coated with silane (3-aminopropyltriethoxysilane) and were dried for 30 min at 37°C. Paraffin was removed with xylene and the sections were rehydrated through a series of graded alcohols. Endogenous peroxidase activity was blocked by incubating the sections in a methanol bath containing 3% hydrogen peroxide for 20 min, followed by washes in distilled water. The sections were initially submitted to heat-induced epitope retrieval using citrate buffer (pH 9.0) in an uncovered pressure cooker (Eterna; Nigro, Araraquara, Brazil). Briefly, the slides were immersed in the buffer solution and the pressure cooker was closed with the safety valve open. Once the saturated steam was released, the safety valve was lowered until full pressurisation was achieved. After 4 min of full pressurisation, the closed pressure cooker was placed under running water for cooling. After removing the lid, the slides were washed in distilled running water and endogenous peroxidase activity was blocked using 10 volumes of 3% H_2_O_2_. After 3 washes (10 min each), the slides were washed in distilled running water and then in phosphate-buffered saline (10 mM; pH 7.4) for 5 min each. The slides were incubated with primary antibody overnight at 8°C.

The following primary antibodies were purchased from Abcam (Cambridge, MA, USA): anti-SPARC antibody, polyclonal rabbit IgG isotype (1:400; ab14174); anti-SPP1 antibody, monoclonal mouse isotype IgG2a (1:400; ab69498), anti-fibronectin antibody, mouse IgG1 isotype (clone IST-9) (1:400; ab6328), anti-integrin vs. primary antibody, mouse IgG2a (clone 10F6) (1:400; ab93943), and anti-integrin vs. antibody, isotype mouse IgG1, clone 272-17E6 (1:400; ab16821).

GS and DFS were defined as the time from an initial anatomopathological diagnosis until the patient’s death and first recurrence, respectively.

### Statistical methods

The Statistical Package for Social Sciences (v18.0) was used for the analysis of data. The significance level was 0.05 or 5%. Kaplan-Meier survival analyses were performed to identify differences in GS and DFS for each category of hypo- or hyperexpression of each marker. The log-rank test was used to verify whether the differences were significant. Stepwise Cox regression models were used to identify the risk factors associated with GS in a multivariate mode and then were used to score the risk of incidence of death for each marker independently, or in association. Logistic regression was used to verify the association between the risk scores for markers associated with GS and TNM classification. Using the variables that were significantly associated in the multivariate analyses for GS and DFS, additive risk scores were established. These scores were assigned for each category of risk (hyper- or hypoexpression, depending on the marker) and these added up to 1. The resulting ordinal variable underwent Cox regression (for GS and DFS) to verify the association between the risk score created and the respective dependent variables.

## Results

### Univariate analyses of GS in relation to the ECM markers studied

**Fig 1:**
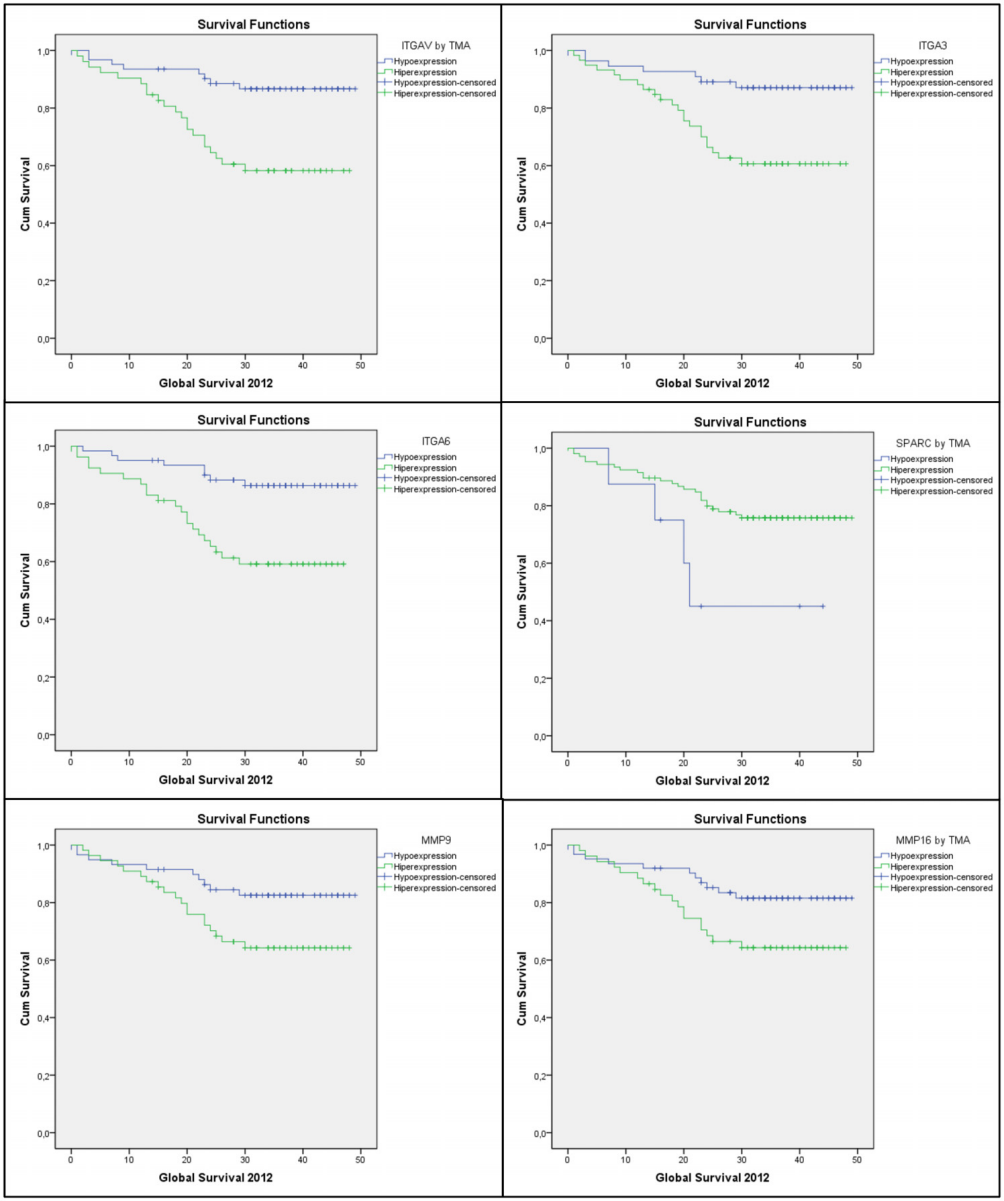
Kaplan-Meier GS curves associated with the hypo- and hyperexpression of ITGAV, ITGA3, ITGA6, SPARC, MMP9, and MMP16.

Table 1 presents the clinical characteristics and the parameters for tumour dissemination for the 114 patients of the present cohort. Significant differences in GS were identified in relation to the expression profiles of ITGAV1, ITGA3, ITGA6, SPARC, MMP9, and MMP16, markers that were detected in the tumour samples examined. For SPARC, hyperexpression was associated with a higher level of survival. In contrast, hyperexpression of the other markers were associated with lower levels of GS. Fig 1 shows the GS curves that were obtained according to the status of each significantly associated marker. Table 2 lists the results of the Kaplan-Meier survival analyses in relation to GS and the expression of the markers studied.

**Table 1:** Clinicopathological characteristics of the 114 patients included in this study.

Variables	n	%
**Median age(years/interval)**	60 (24–83)	
** Age group**		
< 60 years	56	49.1
≥ 60 years	58	50.9
**Location of the primary tumour**		
Right colon	41	36.0
Left colon	41	36.0
Rectum	32	28.0
**Synchronous tumour**		
No	112	98.2
Yes	2	1.8
**Histological type**		
Adenocarcinoma NOS	97	85.1
Mucinous adenocarcinoma	17	14.9
**Grade of differentiation**		
Well-differentiated	9	7.9
Moderately differentiated	91	79.8
Poorly differentiated	14	12.3
Undifferentiated	0	0
**Surgical margin**		
Compromised by malignancy	0	0
Free from malignant neoplasm	114	100
**Site of metastasis**		
Unknow	98	85,9
Liver	9	7.9
Peritoneum	3	2.6
Lungs	2	1.8
Ovary	2	1.8
**TNM Stage**		
I	25	21.9
II	39	34.2
III	34	29.8
IV	16	14.0

**Table 2:** Analysis of GS in relation to of the ECM markers Detected.

Variable	Category of Expression	Average	EP	95% CI		Log-rank χ^2^	Log-rank p-value
				Lower	Upper		
**ITGB5**	Hypo	37.4	2.0	33.4	41.4	3.106	0.078
	Hyper	43.1	1.8	39.5	46.7		
**ITGAV**	Hypo	44.5	1.6	41.4	47.5	11.396	**0.001**
	Hyper	34.8	2.3	30.2	39.3		
**ITGA3**	Hypo	44.5	1.7	41.2	47.7	9.477	**0.002**
	Hyper	35.6	2.2	31.4	39.8		
**ITGA5**	Hypo	42.8	1.8	39.3	46.3	2.484	0.115
	Hyper	37.4	2.1	33.3	41.5		
**ITGA6**	Hypo	44.6	1.5	41.7	47.6	10.845	**0.001**
	Hyper	34.1	2.3	29.7	38.6		
**Fibronectin**	Hypo	40.3	1.5	37.3	43.3	0.147	0.701
	Hyper	38.5	3.1	32.5	44.6		
**SPARC**	Hypo	28.7	5.3	18.2	39.2	4.419	**0.036**
	Hyper	41.0	1.5	38.1	43.8		
**SPP1**	Hypo	37.3	4.4	28.7	45.9	0.036	0.849
	Hyper	40.4	1.5	37.4	43.4		
**VCAM**	Hypo	36.3	2.7	31.0	41.5	3.203	0.073
	Hyper	41.9	1.6	38.7	45.2		
**MMP1**	Hypo	41.5	1.6	38.4	44.6	2.773	0.096
	Hyper	35.1	3.2	28.9	41.3		
**MMP2**	Hypo	42.8	1.8	39.3	46.3	2.484	0.115
	Hyper	37.4	2.1	33.3	41.5		
**MMP9**	Hypo	43.0	1.8	39.5	46.5	4.507	**0.034**
	Hyper	36.8	2.2	32.6	41.0		
**MMP11**	Hypo	41.3	2.3	36.8	45.9	0.365	0.546
	Hyper	39.0	1.8	35.5	42.5		
**MMP16**	Hypo	42.9	1.8	39.4	46.3	4.292	**0.038**
	Hyper	36.6	2.2	32.2	41.0		
**COL6A2**	Hypo	36.6	2.6	31.4	41.7	2.806	0.094
	Hyper	41.8	1.7	38.6	45.1		

GS: global survival.; EP: equal-precision confidence bands.; CI: confidence interval.

### Multivariate analyses of GS in relation to the ECM markers studied

The variables significantly associated with GS were analysed in a stepwise Cox regression model. Table 3 shows the results of the multivariate analysis with GS used as the dependent variable and the associated markers used as independent variables.

**Table 3:** Stepwise Cox regression models for GS using hyperexpression of the markers detected.

Model	GS	p-value	HR	95% CI	
				Lower	Upper
1	ITGAV	0.043	3.141	1.038	9.506
	ITGA3	0.310	1.846	0.565	6.029
	ITGA6	0.010	3.144	1.308	7.553
	SPARC	0.446	0.643	0.206	2.002
	MMP9	0.512	0.474	0.051	4.396
	MMP16	0.933	1.091	0.143	8.323
2	ITGAV	0.043	3.135	1.036	9.484
	ITGA3	0.311	1.843	0.565	6.019
	ITGA6	0.010	3.152	1.315	7.555
	SPARC	0.442	0.641	0.206	1.991
	MMP9	0.223	0.516	0.178	1.495
3	ITGAV	0.033	3.272	1.098	9.748
	ITGA3	0.253	1.971	0.616	6.307
	ITGA6	0.005	3.381	1.446	7.905
	MMP9	0.164	0.469	0.162	1.362
4	ITGAV	0.004	4.189	1.571	11.172
	ITGA6	0.004	3.444	1.475	8.042
	MMP9	0.320	0.618	0.240	1.594
**5**	**ITGAV**	**0.005**	**3.209**	**1.412**	**7.293**
	**ITGA6**	**0.007**	**3.105**	**1.367**	**7.055**

GS: global survival; HR: hazard ratio; CI: confidence interval.

Model 5 shows that the variables ITGAV and ITGA6 were significantly associated with GS. Furthermore, hyperexpression of these markers was associated with a greater risk of death during the period studied (hazard ratio (HR) = 3209,; 95% confidence interval (CI) = 1.412–7.293, p = 0.005 and HR = 3.105, 95% CI = 1.367–7.055, p = 0.007, respectively).

### Univariate analyses of DFS in relation to the ECM markers studied

**Fig 2:**
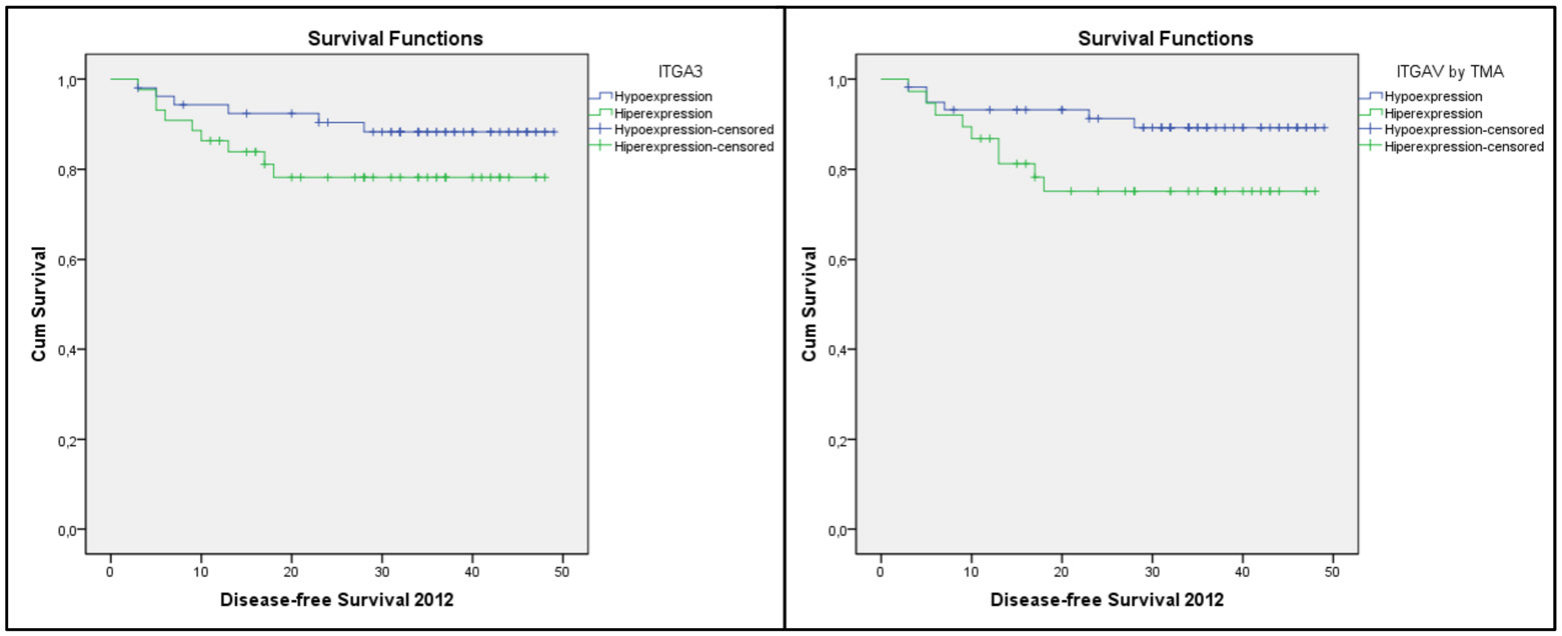
Kaplan-Meier DFS curves associated with the hypo- and hyperexpression of ITGA3 and ITGAV.

Table 4 shows the results for the Kaplan-Meier survival analysis performed for DFS in relation to the expression of the markers studied. Significant differences in DFS were observed in relation to expression of ITGAV and ITGA3, with hyperexpression being associated with lower levels of DFS (Fig 2). Parameters for the SPARC marker were not calculated, as there were no recurrence events in the hypoexpression group.

**Table 4:** Analysis of DFS in relation to expression of the ECM markers detected.

Variable	Category of expression	Average	EP	95% CI		Log-rank χ^2^	Log-rank p-value
				Lower	Upper		
**ITGB5**	Hypo	42.152	1.815	38.595	45.708	2.156	0.142
	Hyper	38.193	2.355	33.578	42.808		
**ITGAV**	Hypo	43.979	1.593	40.858	47.101	8.296	**0.004**
	Hyper	34.605	2.713	29.286	39.923		
**ITGA3**	Hypo	44.929	1.499	41.992	47.867	10.197	**0.001**
	Hyper	34.47	2.563	29.446	39.493		
**ITGA5**	Hypo	39.105	2.287	34.622	43.588	0.820	0.365
	Hyper	41.159	1.962	37.313	45.005		
**ITGA6**	Hypo	40.433	1.841	36.824	44.041	0.280	0.597
	Hyper	39.28	2.473	34.434	44.127		
**Fibronectin**	Hypo	39.063	1.787	35.56	42.567	0.775	0.379
	Hyper	42.583	2.624	37.44	47.726		
**SPARC**	Hypo	-	-	-	-	1.041	0.308
	Hyper	-	-	-	-		
**SPP1**	Hypo	39.146	4.39	30.542	47.749	0.019	0.891
	Hyper	40.449	1.612	37.29	43.607		
**VCAM**	Hypo	39.65	2.768	34.224	45.075	0.014	0.905
	Hyper	40.57	1.815	37.013	44.126		
**MMP1**	Hypo	41.33	1.676	38.045	44.614	0.910	0.340
	Hyper	36.589	3.267	30.186	42.992		
**MMP2**	Hypo	39.105	2.287	34.622	43.588	0.820	0.365
	Hyper	41.159	1.962	37.313	45.005		
**MMP9**	Hypo	43.34	1.662	40.082	46.598	3.664	0.056
	Hyper	36.201	2.603	31.099	41.303		
**MMP11**	Hypo	39.304	2.506	34.392	44.216	0.731	0.393
	Hyper	40.582	1.855	36.946	44.218		
**MMP16**	Hypo	42.482	1.72	39.111	45.853	1.759	0.185
	Hyper	36.867	2.685	31.603	42.13		
**TBS1L**	Hypo	39.667	2.139	35.474	43.86	0.760	0.383
	Hyper	40.64	2.113	36.498	44.782		
**COL6A2**	Hypo	39.939	2.67	34.706	45.172	< 0.001	0.983
	Hyper	40.463	1.839	36.858	44.067		

DFS: disease-free survival; ECM: extracellular matrix; EP: equal-precision confidence bands.; CI: confidence interval.

### Multivariate analyses of DFS in relation to the ECM markers studied

Model 2 of Table 5 shows that only ITGA3 was significantly associated with DFS. Hyperexpression of this marker was associated with a higher risk of recurrence during the period studied (HR = 3.806, 95% CI = 1.573–9.209, p = 0.003). Analyses of the score for DFS were not performed since only one gene (ITGA3) maintained a significant association.

**Table 5:** Cox regression model for DFS (significant variables only).

Model	Category of expression	p-value	OR	95% CI	
				Lower	Upper
1	ITGAV (Hyper)	0.387	1.615	0.544	4.792
	ITGA3 (Hyper)	0.088	2.748	0.861	8.775
**2**	**ITGA3 (Hyper)**	**0.003**	**3.806**	**1.573**	**9.209**

DFS: disease-free survival; OR: odds ratio; CI: confidence interval.

### Calculation of risk scores based on the results of the multivariate model and a comparison of the outcomes

**Fig 3:**
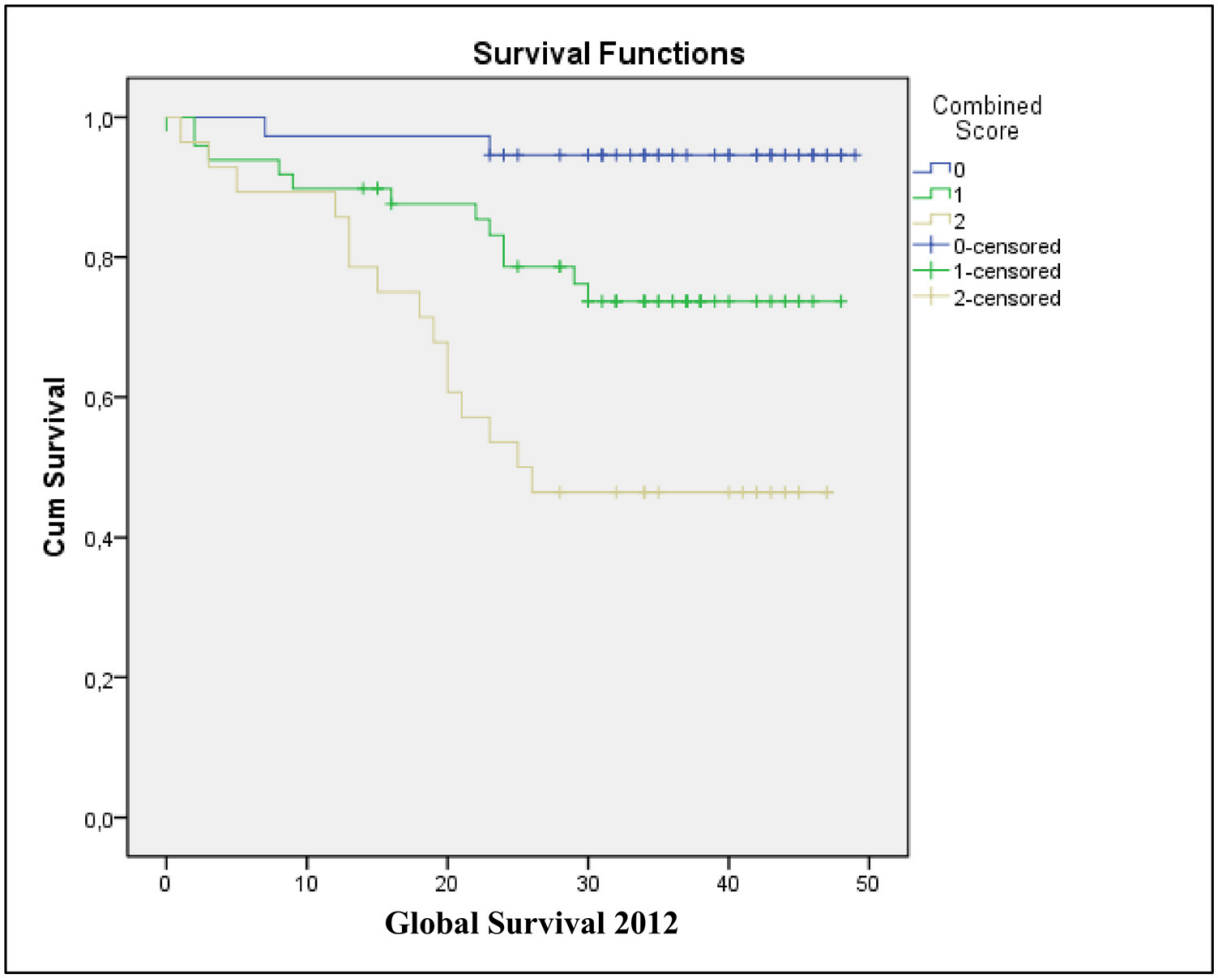
Kaplan-Meier GS curves for each category of the combined score.

A risk scoring system was created based on the results of the multivariate analysis of the significant variables to verify whether the presence of 0, 1, or 2 of the associated markers affected the risk of death (Table 6). For each marker of risk that was hyperexpressed, one unit was added to the score. After establishing this scale, the results (ordinal categorical variables) were compared with GS using the log-rank test and Cox regression analysis (Fig 3, Table 6). A pattern of association was observed between the number of hyperexpressing markers and GS (Table 7), with a good association observed between the presence of one or two markers and the ability to predict GS (Table 7).

**Table 6:** Analysis of GS in relation to scoring based on hyperexpression of ITGAV and ITGA6.

Number of hyperexpressed markers	Mean survival	EP	95% CI	
			Lower	Upper
0	47.162	1.300	44.613	49.711
1	39.717	2.166	35.471	43.964
2	30.179	3.137	24.030	36.327

GS: global survival.; EP: equal-precision confidence bands.; CI: confidence interval.

**Table 7:** Cox regression analysis using the combined risk score of ITGAV and ITGA6 overexpression as an independent variable and GS as a dependent variable.

Global Survival	p-value	HR	95% CI	
			Lower	Upper
One hyperexpressed marker	**0.030**	5.262	1.177	23.516
Two hyperexpressed markers	**0.001**	13.463	3.072	59.002

GS: global survival.; HR: hazard ratio.; CI: confidence interval.

### Calculation of the association between prognostic score and TNM

The scoring system that was developed based on the detection of none, one, or both of the ITGAV and ITGA6 markers being hyperexpressed was found to have the greatest effect on GS. We wanted to verify an association between this scoring system and TNM through logistic regression. Table 8 lists the cases with hyperexpression of both markers, and they were associated with a 33.5 fold higher chance of belonging to the TIII+IV TNM category. However, since there were no patients who were both stage TIII+TIV and that hyperexpressed any of the markers assayed, the cases with hyperexpression of 0 genes and the cases with hyperexpression of 1 gene were combined and were compared with the cases with hyperexpression of 2 genes (Table 9).

**Table 8:** Logistic regression using the combined risk score (hyperexpression of ITGAV and ITGA6–0 or 1 hyperexpressing marker versus 2 hyperexpressing markers) as an independent variable and TNM as a dependent variable.

TNM	p-value	OR	95% CI	
			Lower	Upper
Two hyperexpressing markers (vs. 0 or 1)	**< 0.001**	33.583	7.393	152.546

OR: odds ratio.; CI: confidence interval.

**Table 9:** Analysis of GS in relation to TNM stage.

Stage	Mean survival	EP	95% CI	
			Lower	Upper
Stage I+II	44,898	1,525	41,909	47,886
Stage III+IV	33,857	2,336	29,279	38,435

GS: global survival.; EP: equal-precision confidence bands.; CI: confidence interval.

**Fig 4:**
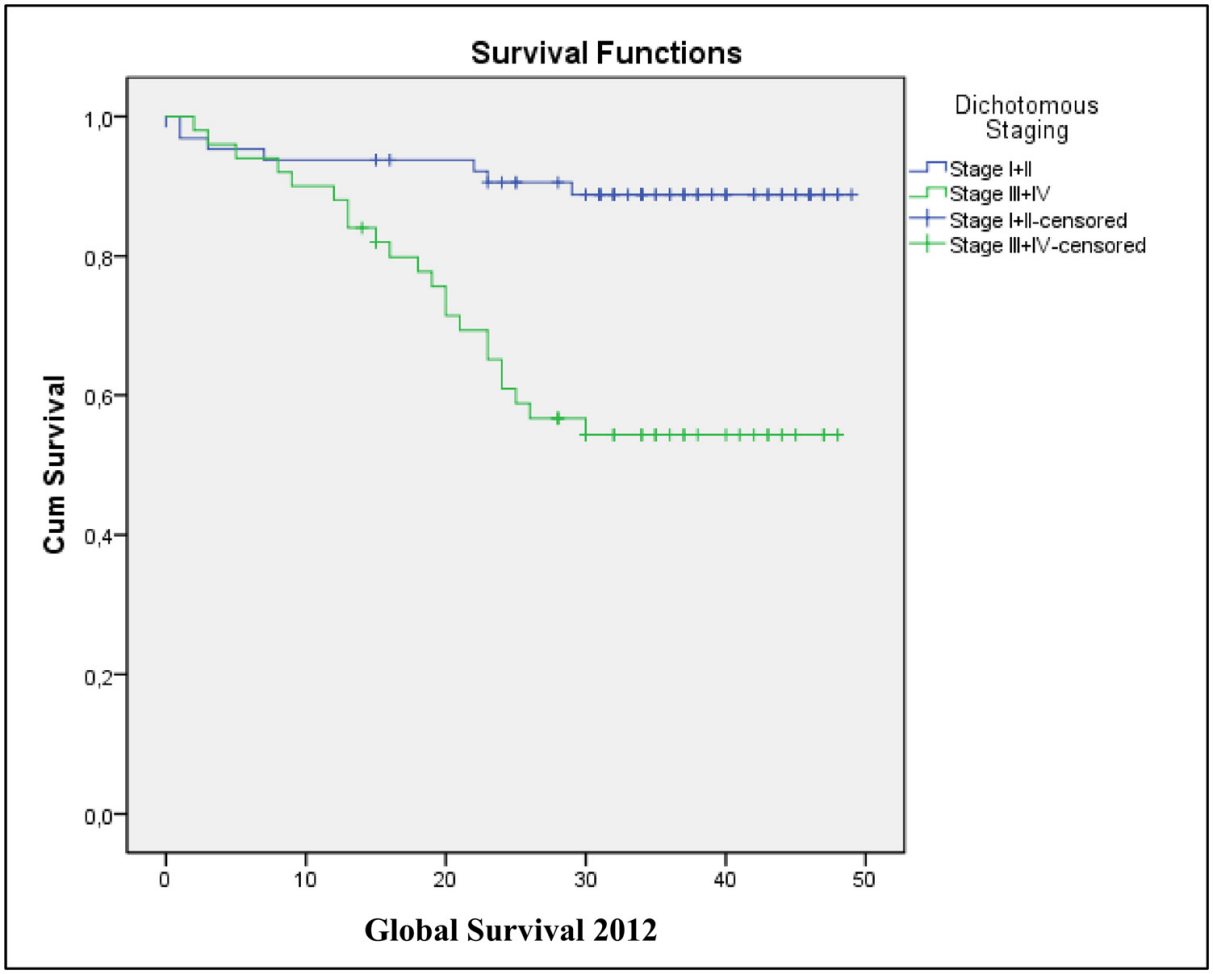
Kaplan-Meier GS curves according to tumor stage.

Finally, to verify whether TNM stage can serve as a predictor of survival, a survival analysis was performed in relation to TNM category (I+II vs. III+IV) (Table 10, Fig 4). Table 10 verifies that patients with a stage III+IV tumour had a 4.838-fold higher risk of death (HR = 4.838).

**Table 10:** Cox regression analysis using TNM stage as the independent variable and GS as the dependent variable.

Global Survival	p-value	HR	95% CI	
			Lower	Upper
**Stage (III+IV)**	**< 0.001**	4.838	2.063	11.349

HR: hazard ratio.; CI: confidence interval.

## Discussion

The capacity for a malignant tumour to migrate and spread to other tissues and organs is determined by various molecules, including proteolytic enzymes, adhesion molecules, cell receptors, cytokines, and growth factors. This involves a complex system of signal transduction to the cell nucleus via membrane and intracellular molecule receptors that transmit feedback from the ECM, thereby resulting in the activation and synthesis of transcription factors for different genes. [21] Accumulating evidence indicates that genetic changes are responsible for the rapid progression of various types of malignant tumours from the initial stages of disease to advanced stages of disease. It is hypothesised that this process is initiated by molecules that anomalously signal to activate genes that affect dissemination and metastasis. The identification of such molecules and their altered structures, as well as their association with clinical stages and pathology, might clarify the mechanisms involved in carcinogenesis, and therefore, the groups of genes involved in this process.

According to Koivisto et al. (2000), the ECM can influence the behavior of a neoplasm by enhancing tumour cell proliferation, progression, and invasion. These interactions are mediated by integrins which have been shown to play an important role in the development of tumour invasion and metastasis. Degradation of the ECM has also been reported to occur via the action of proteolytic enzymes that are mainly produced by tumour cells, yet can also be activated by stromal fibroblasts. [22]

In the present study, interactions between patient tumour cells and the ECM were characterized based on the identification of hypo- or hyperexpressed genes, with the hypothesis that these genes could be important for predicting prognosis and patient survival. To profile gene expression, the Super Array Kit (PAHS-031A-24, AMBRIEX) was used to detect the expression levels of ECM and cell adhesion molecules (n = 84) that are important for cell-cell and cell-matrix interactions. This set of genes included proteins from the ECM that represent important constituents of the basal membrane. Real-time PCR provided a rapid, simple, and reliable method for analyzing the expression of a group of proteins involved in the process of tumour progression and dissemination of colorectal adenocarcinoma in its various phases of staging. In combination with immunohistochemical data, the identification of associations between the parameters of tumour progression and the expression profile of hypo- or hyperexpressed genes was anticipated to provide insight into tumour progression and dissemination. The second objective of the present study was to build a mathematical model or a scoring system that would allow for the identification (even in the pre-operative period with the analysis of endoscopic biopsies) of patients with a poor prognosis. If reliable, this model would allow differentiated therapies to be selected for patients based on a better or worse prognosis.

Integrins are cell surface heterodimer receptors that are composed of α and β transmembrane subunits. Each subunit includes a large extracellular transmembrane domain and an intracellular domain. [23] Cell adhesion interactions play an important role during normal physiological processes such as embryonic development and tissue healing, and also during pathological processes such as cancer. [24] Regarding the latter, Zhang et. al (2011) demonstrated that integrins play a role in multiple steps of carcinogenesis, including cell dissemination, cell invasion of adjacent tissues, and cell survival. They also play a regulatory role in cell survival and apoptosis, thereby promoting the growth of tumour tissue and the process of metastasis. Correspondingly, antineoplastic therapeutic potential has been identified for various integrin antagonists, such as α5β1, αVβ3, and αVβ5, and these are in an experimental phase of study. [25] Higher expression of these integrins is associated with greater migration and invasion of cancerous cells, and is also associated with increased resistance to antineoplastic drugs. In contrast, lower levels of expression of certain integrins, such as α2β1 and α1β1, by tumour cells can favor cell diffusion. In addition to changes in expression, changes in the function of these integrins has been shown to play a fundamental role in cancer progression. [19,26]

In the present study, univariate analyses showed a statistically significant association between the hyperexpression of integrins, ITGAV, ITGA3, and ITGA6, and the MMP9 and MMP11 genes. In addition, hypoexpression of the SPARC gene was associated with a reduction in the GS of CRC cancer patients (Table 2, Figs 1–4). However, in the multivariate model, only hyperexpression of the ITGAV and ITGA6 integrins was associated with a greater risk of death during the period studied (HR = 3.209, 95% CI = 1.412–7.293, p = 0.005 and HR = 3.105, 95% CI = 1.367–7.055, p = 0.007, respectively) (Table 3).

The α5β1, α6β4, αVβ3, and αvβ6 integrins have been widely studied in malignant tumours, and their levels of expression have correlated with the progression of various types of tumours. [27–28] Other studies have identified integrin expression as factors in the dissemination and prognosis of CRC patients. For example, hepatic dissemination has been found to depend on interactions between the αVβ6 integrins expressed by tumour cells and the fibronectin marker of hepatic microvasculature. [29] In addition, liver metastases have been associated with the β1 integrin [23], while advanced clinical stages and venous and perineural invasion have been associated with hyperexpression of αV integrins. [19,20,30] For pulmonary metastases expressing β1 and β2 integrins (e.g., α2β1, α4β1, α5β1, and αLbα2) [31], a poorer prognosis was associated with hyperexpression of αVβ3 and the αV integrins. [32,33]

In a previous publication, our group identified an association between hyperexpression of the ITGAV gene in tumours and the presence of perineural invasion in colorectal tumours. [19] Correspondingly, histochemical analyses showed that this marker was hyperexpressed in 100% of patients with distant metastasis, and was hyperexpressed in 36.7% of patients without distant metastasis. [19] In addition, the multivariate analysis showed that tumours with lymph node metastasis had a 108-fold greater chance of hyperexpressing ITGAV than the tumours without lymph node metastasis. [19]

Increased expression of ITAG6 was also detected in the presence of venous invasion compared with the absence of venous invasion (p < 0.04). [19] These findings suggest that increased expression of this integrin promotes tumour dissemination. Similarly, in comparing the scores from the histological analyses performed in the present study, correlations between tumour type (mucinous or adenocarcinomas NOS) and the ITGA5 and ITGA6 integrins were observed (p < 0.001). A higher percentage of mucinous type histology samples also received a score of 2 more often than the adenocarcinoma NOS samples.

The a6 integrin regulates various cell functions, including the induction of cell invasion, migration, tumour cells, and tumour progression. [34] On the other hand, O'Connor et al. (2000) reported that lower levels of α6 expression correlated with an increased migratory and invasive potential for colon cancer cells. [35] We hypothesise that the processes that enable a tumour cell to escape cell and tissue containment mechanisms, and that also facilitate its growth, dissemination, and consequent colonisation of secondary tissues, arise from the activation (hypo- or hyperexpression) of multiple genes simultaneously, and these act to overcome a host’s protective barriers. Thus, the objective of the present study was to develop a multivariate mathematical model of the hypo- or hyperexpression of genes related to neoplastic dissemination, in order to provide the most accurate wide-scale mapping of the processes that occur simultaneously during tumour progression and that affect prognosis. The final multivariate prognostic model achieved was composed of the ITGAV and ITGA6 integrins, which were found to correlate with GS, thereby suggesting that hyperexpression of these genes represents an independent action in reducing the survival of colorectal tumour patients. The findings of our multivariate model also correlate with the results of previous univariate studies that have described an association between the ITGAV integrin and the presence of distant metastases, lymph node metastases, and perineural invasion. [20] In addition, expression of the ITAG6 integrin has been associated with a mucinous histological type (which has a poorer prognosis) and with venous invasion. [19] Based on these results, we hypothesise that these two integrins act on different processes and that they can act independently, or collectively, to promote tumour dissemination and progression, thereby compromising patient prognosis (e.g., GS).

In the univariate analyses performed for DFS in the present study, hyperexpression of the ITGAV and ITGA3 integrins were found to correlate with DFS (Table 4). However, when we built a multivariate model, only ITGA3 exhibited a significant association with DFS (Table 5). Therefore, hyperexpression of this marker was associated with a greater risk for recurrence during the period studied (HR = 3.806, 95% CI = 1.573–9.209, p = 0.003). In previous studies, the ITGA3 integrin has shown an association with lymph node or distant metastasis (TNM III and IV) [19,36], which is consistent with the present findings where more advanced TNM stages were found to correlate with a lower expectation for DFS. To our knowledge, this is the first study to develop a multivariate mathematical model for GS and DFS based on integrin gene expression that is capable of predicting the prognosis for colorectal tumours, even during the postoperative period.

To facilitate the clinical application of the multivariate prognostic prediction findings described in the present study using a multivariate Cox regression model, a scoring system was established based on the number of hyperexpressed markers detected in the tumor tissue samples. This system was found to significantly correlate with the GS curves for each category (0, 1, and 2 for the ITGAV and ITGA6 markers; Table 6), as well as with the more advanced TNM stages (Table 9). These results show that patients hyperexpressing both of these genes had a 33.583-fold greater risk of belonging to TNM categories TIII + TIV than TNM categories I + II (Table 9). To certify that the present analyses of GS correlated with dichotomised TNM stages (e.g., TI + T2 vs. T3 + T4), a Cox regression was performed (Table 10). It was observed that TNM stage III + IV patients had a 4.838-fold greater risk of death than the TNM stage I + II patients.

In conclusion, a multivariate mathematical model was generated that demonstrated an association between hyperexpression of the integrins, ITGAV and ITGA6, and GS, and also between the ITGA3 integrin and DFS, in patients with colorectal tumours. A risk scoring system was also established based on the detection of none, one, or two overexpressed markers (ITGAV and ITGA6), and this system accurately correlated with the GS curves obtained for the present cohort.

## Abbreviations

CRC: colorectal cancer
GS: Global Survival
DFS: Disease-free Survival
ECM: extracellular matrix
ITGAV: Integrin Alpha V
ITGA3C: Integrin Alpha 3
ITGA6: Integrin Alpha 6
MMP1: Matrix Metallopeptidase 1
MMP16: Matrix Metallopeptidase 16
COL6A2: Type VI Collagen gene
THBS1: Thrombospondin 1
VCAM-1: Vascular Cell Adhesion Molecule 1
SPARC: SPARC gene
EGFR: Epidermal Growth Factor Receptor
VEGF: Vascular Endothelial Growth Factor
